# Between violation and competent care—Lived experiences of dependency on care in the ICU

**DOI:** 10.3402/qhw.v10.26603

**Published:** 2015-03-11

**Authors:** Kristina Lykkegaard, Charlotte Delmar

**Affiliations:** 1 Intensive Care Unit, Aalborg University Hospital, Aalborg, Denmark; 2 Section for Nursing, Department of Public Health, Faculty of Health Sciences, Aarhus University, Aarhus, Denmark; 3 University College Diakonova, Oslo, Norway

**Keywords:** Dependency, relationship, intensive care, ambivalence, violation, gratitude

## Abstract

This study explores the perceived meaning of dependency on care as experienced by intensive care patients. Research from non-intensive settings shows that dependency is often experienced negatively, but literature on the subject experienced by patients in the ICU is sparse. The study is based on in-depth qualitative semi-structured interviews of lived experience with three former patients admitted to an intensive care unit at a Danish university hospital. The in-depth interviews have been characterized as narratives. The main inspiration for the analysis method is Ricoeur's phenomenological hermeneutical interpretation theory. The study has found that dependency is experienced as difficult, and the relationship with the nurses seems to be ambivalent. The good relationship is experienced to make dependency easier, whereas negative experiences make it harder to cope with dependency. The participants deal with dependency by accepting negative experiences in gratitude for having recovered from critical illness. The findings might be influenced by studies being conducted in a western country setting where independence is valued. They can be used as means of reflection on nursing practice and matters such as communication and patient participation.

The aim of this study is to explore the perceived meaning of being dependent on care as experienced by intensive care patients. Patients admitted to intensive care consist of a heterogeneous group admitted for very different reasons. These patients share a high degree of dependency on others as their ability to perform self-care is reduced. The perceived meaning of dependency is unfolded in relationships because human existence is a mutual dependency on each other (Løgstrup, 1996/1971). The collaboration between nurses and patients is therefore of interest. Dependency, though, is not typically verbalized by nurses, either as part of the care actions, nor in the collaboration with colleagues (Strandberg & Jansson, 2003). In other words, healthcare staffs are challenged to know how dependency becomes a lived experience by patients, although dependency is often associated with negative feelings, powerlessness, frailty, and vulnerability.

## Theoretical and empirical background

### Dependency in an ontological and cultural perspective

According to Henriksen and Vetlesen (2000), dependency determines what it means to be a human being. Dependency is linked to a resulting vulnerability meaning that the human being is referred to the care of others, as the human being is not a self-sufficient self, but always in relationships with other human beings. In this referral there is an inherent risk of not being seen and the caring needs of the individual are not met. Dependency thus contributes to making life frail. Dependency on others is constituted by our lives, but it is often in connection with severe illness that we become conscious of this dependency on others. It is when serious illness marks you, you become aware of things that have functioned previously.

Dependency on others is often associated with negative feelings and powerlessness and that is a value-laden concept. This can be understood from a cultural–historical perspective as autonomy in the western culture is considered an overall value. Autonomy means to be self-governing and self-determining. This interpretation of the concept of autonomy comes from the moral philosopher Kant (1724–1804) and is still leading today. To be self-sufficient financially and in relation to work as a cultural ideal can create a basis for the thought that as human beings we are independent. To be independent becomes an ideal demonstrating freedom that can be won by the human being. Dependency is considered something you can grow out of or detach yourself from. Thus, there is a tension between dependency as a basic human condition and the cultural ideal of individual freedom and independency (Henriksen & Vetlesen, 2000).

### Dependency on care in the ICU

The literature review showed that the majority of studies investigate dependency from an organizational perspective. They focus on how dependency can be estimated to assess the optimal nurse–patient ratio, the effect of different classification systems for this assessment, or an estimation of the workload of nurses (Adomat & Hewison, 2004; Adomat & Hicks, 2003; Donoghue, Decker, Mitten-Lewis, & Blay, 2001; Large, Nattrass, & Simpson, 1991; Wardle, 1997).

Three articles discuss different aspects of dependency on care from a patient perspective. Griffin (1982) discussed how the self of critically ill patients is threatened when they are forced to be dependent on others. Forced dependency and loss of control are described as significant stressors in the intensive care patient; some patients described the feeling of lack of control and helplessness as more threatening than the threat of death. The article is rather old, and it is uncertain whether it is based on actual research as it has not been possible to procure the sources on which the article is based.

Two newer studies from the intensive care setting describe dependency, but none of the studies have particularly focused on dependency. McKinley, Nagy, Stein-Parbury, Bramwell, and Hudson (2002) studied the experience of being a critically ill patient at an intensive care unit. The study found that vulnerability is the concept that best characterizes the intensive care patient. When needs are met and the care is individual, patients experience a sense of security in vulnerability. Almerud, Alapack, Fridlund, and Ekebergh (2007) corroborated these findings. The incomprehensible environment and technology limit the patients. Control, influence, and freedom disappear and patients leave themselves to others; this leads to contradictory feelings of security and vulnerability. Dependency on others and technology are described as suffering where the patients have no other choice but to surrender to machines and routines. The study showed that patients try to be good patients by adapting to the expectations of the system.

Two studies focusing on people receiving respirator treatment in their home show that the correct help contributed to an experience of freedom (Dreyer, Steffensen, & Pedersen, 2010; Martinsen & Dreyer, 2012). However, there are no empirical studies focusing on experienced dependency in the ICU. As patients are increasingly awake during the stay at the intensive care unit as a consequence of altered sedation practices, it can be assumed that patients are more conscious of dependency on others compared to previously. It thus seems relevant to focus on the meaning intensive care patients attach to being dependent on care.

## Design and methods

The study is based on three in-depth interviews following the participants’ narratives. With a phenomenological hermeneutic approach, the lived experiences were collected thorough semi-structured questions (Brinkmann & Tanggaard, 2010; Kvale & Brinkmann, 2009) and the analytical method is inspired by Lindseth and Norberg (2004). Their method has been used in several studies and applied to health care research and human studies when it is important to obtain knowledge of the meaning of lived experience. The main inspiration for developing their method is Ricoeur's phenomenological hermeneutical interpretation theory. The intention with the method is to improve understanding of a phenomenon. It can be used when studying phenomena such as dependency (Dreyer & Pedersen, 2009). Through the patients lived experiences and the resulting narratives, the researcher tries to obtain a possible meaning of being dependent on care. The researcher must be aware of his pre-understanding, especially because interview knowledge is an asymmetrical power relation as the interview is defined by the researcher. It is important that the researcher reflects on the significance of this power and pre-understanding in relation to the knowledge produced (Kvale & Brinkmann, 2009, pp. 50–52). During the interviews it has been attempted to follow the participants’ narratives and let them finish talking before asking any elaborating questions showing respect for the participants’ lived experiences and not the pre-understanding of the researcher. Also during the analysis the texts have been carefully considered if interpretations are rooted in the participants’ narratives. To reduce bracketing the analysis and interpretation process were consecutively discussed with two very experienced researchers. In addition, the interviewer had knowledge of the department where the informants were hospitalized, whereby the pre-understanding might influence the findings. However, it was seen as an advantage to be aware of the context in which the informants’ narratives emerged.

### Participants

A purposeful sample was made (Polit & Beck, 2010). Eight persons were contacted by the head nurse at first; three persons agreed to participate (Table I). The participants were recruited from a high-technology intensive care unit at a larger Danish university hospital receiving medical and surgical patients. It is known that many intensive care patients have recall problems. Prolonged admission and respirator treatment influence the ability to recall the stay and the respirator treatment including the experiences attached to nursing care (Bergbom-Engberg, Hallenberg, Wickstrom, & Haljamae, 1988; Capuzzo et al., 2001). To minimize some of the recall problems, inclusion criteria specified that patients must have been discharged from the intensive care unit within the last 6 to 12 months and have received respirator treatment for 1 week or longer. Because of the recall problems it was very difficult to recruit participants.

**Table I:** Description of the three participants.

•	Participant 1: 27-year-old female admitted to the intensive care unit due to surgical complications. The admission lasted 3 weeks. The woman subsequently stayed for a prolonged period of time at a surgical bed ward and has needed help from the family after discharge from the hospital. She was interviewed 1 year after admission to the intensive care unit and at this time she had resumed her job.
•	Participant 2: 78-year-old woman admitted to the intensive care unit due to a complicated postoperative course. Moreover, the woman had a known medical problem, which had made the treatment difficult. She was admitted for 3 months and subsequently she participated in prolonged rehabilitation; the total length of hospital admission was approximately 6 months. She was interviewed 3 months after discharge from the intensive care unit where she has just come home. She still needs help in the home; the help is provided by family and the professional system. During the stay at the intensive care unit the woman has been transferred to a smaller hospital with a smaller intensive care unit and her recollections are primarily from the unit. The woman is a pensioner.
•	Participant 3: 56-year-old male admitted to the intensive care unit due to a medical problem. The stay lasted 3 weeks and subsequently he has been admitted to a medical ward. He is interviewed approximately 3 months after discharge from the intensive care unit. At this time he is temporarily staying at a nursing home. He has been an early retirement pensioner for 15 years.

Adults above 18 years interested in telling about their experiences were selected. We assumed that there would be other experiences of dependency among children and adolescents. Persons who could speak and understand Danish and had a Danish culture participated. This seems relevant, as dependency is considered a value in the western society. Finally, participants were interviewed after discharge considering ethical issues concerning their well-being, research has shown that intensive care patients are strongly affected emotionally by the intensive care experience during their stays and after discharge to another ward (McKinney & Deeny, 2002).

Patients with signs of posttraumatic stress or patients who were mentally unstable during admission to the intensive care unit did not participate. The head nurse was in telephone contact with former patients who complied with the criteria. Participants were briefly informed about the study by the head nurse and if they were interested, a letter was sent with additional information. Subsequently, participants were contacted by telephone for confirmation of participation and to make an appointment for the time and place of the interview. The written material suggested that interviews take place in the participant's home, but each participant made his or her own decision.

The remaining five were all males; two had no recollection of the stay at the intensive care unit, one declined due to lack of time, one never replied, and one was in such a poor state that participation was not possible according to the spouse. Several patients who, according to the department nurse, were candidates for the study died after discharge from the intensive care unit. Thus, the criteria for discharge were changed to between 3 and 12 months.

Participants had been used to managing on their own before the admission to hospital. During the stay at the intensive care unit they needed comprehensive help “for everything.” In time things developed, especially for participant 2, who said that she was able to eat by herself during the last course of the admission. Participants have had difficulties recalling the time at the intensive care unit and to make coherent narratives. Participant 3 had difficulty distinguishing dreams from reality and episodes from the bed ward and the intensive care unit. It was possible to exclude parts of the interview due to knowledge of the special characteristics of the intensive care unit. It did not have any immediate consequence for the number of recollections when participants were interviewed; this is in line with research in the area (Löf, Berggren, & Ahlström, 2006).

### Materiel and data analysis

The interview guide contained the themes dependency in relation to understanding of self, dependency in relation to time, bodily experiences linked to dependency, and experiences in relation to the nurse. Questions linked to the themes were, for example: Tell me about what it meant for you to be dependent on others help (thoughts, feelings), Did your perceived experience changed during the stay at the ICU?, How did you feel your body? Tell me about an important situation related to the collaboration with a nurse. Themes and questions were based on the literature review. An analysis of the concept “care dependency” (Boggatz, Dijkstra, Lohrmann, & Dassen, 2007) inspired the questions as they recommend that the empirical studies should include questions about the patients’ functional limitations.

The guide functioned as an indicator. Above all, it was the patients’ narratives and experiences that were followed. The interviews lasted between 45 and 60 min and were recorded on an audio file and transcribed verbatim and decontextualized to a text.

The findings appeared through analysis and interpretation of the text, which was carried out on three levels: a naive reading, a structural analysis, and an interpreted whole which can increase, change, or broaden the understanding. This means that it is a continuous process from what is said to what is talked about to which the meaning of what is said can refer. In the naive reading, the text was read several times to grasp its meaning as a whole (Lindseth & Norberg, 2004). This demanded a phenomenological approach where the researcher is open to what the text says and become influenced and moved by it. In this study, the naïve reading and the immediate impressions in relation to “what is said” were, for example, “I was just a body lying there,” and “I will not treat other people in this way,” and “right there I was walking on air.” The naive reading is characterized by guessing which is inherent in the text, it will subsequently be validated or rejected in the structural analysis.

The structural analysis searches for themes, threads of meaning penetrating the whole text or parts of it. These meaning units were read and reflected upon in relation to the naive reading. Subsequently they were condensed which means that the essence of each meaning unit is expressed as precisely as possible using everyday language. Reflections were made on the similarities and differences of the meaning units and a further condensation is made into themes and possibly sub-themes. Finally, reflections were made on the themes in relation to the naive reading. If this cannot be validated by the themes, a new naive reading is made again and a subsequent structural analysis. This process continues until the naive reading can be validated (Lindseth & Norberg, 2004). The reading has been challenged in discourse with each other and with other health care providers. The final themes are the researchers’ interpretation of what the said refers to and the quotation provides the justification hereof. Table II is an example of the structural analysis with other examples than outlined in the section of the findings.

**Table II:** An example of the structural analysis.

Meaning unit	Condensation	Sub-theme	Theme
“The worst part of being dependent on others is that they had to fiddle with me all the time, touch and feel. I am a very private person” (Participant 1)	Care is experienced as instrumental and impersonal	Being dependent on care can mean you are violated	The relationship to the care staff is ambivalent
“The needs are met and in many situations without asking” (Participant 1) “I have talked with the nurses when something is wrong with the care” (Participant 2) “The relationship with the nurses is important, Then you have a personal relationship instead of authorities in uniforms” (Participant 1)	Caring needs are met without asking A sense of community Personal relationships are important	The relation to staff is personal and caring	The relationship to the care staff is ambivalent
“well you, you survive right? (Participant 3) It is okay to be treated as a physical body because you have survived (Participant 1) “I was angry because the staff couldn't understand what I was saying … but no complains” (Participant 3)	The gratitude of having survived An element of humbleness in the gratitude	There is a deep gratitude linked to dependency	The relationship to the care staff is ambivalent

The themes are reflected on with literature texts in the discussion section with the goal of interpreting the text as a whole and arriving at a comprehensive understanding of being a dependent intensive patient.

## Ethical considerations

According to the national ethics committee, an ethical approval was not necessary, as the study did not include biomedical aspects. The study was made in accordance with the ethical guidelines for nursing research in the Nordic countries (Nordic Nurses’ Federation [NNF], 2003). The study was approved by the Danish Data Protection Agency.

All information on participants was dealt with confidentially and audiotapes and interview transcription files were given an ID number and stored separately from sensitive personal data. Prior to each interview, a written and oral informed consent was obtained and it was made clear that participation was voluntary and it was possible to leave the study at any time without further explanation. The participants were also informed that they would be anonymous and that information would be handled confidentially. All participants signed a consent form.

During the interviews the participants’ narratives were listened to and they finished talking before asking any elaborating questions, showing respect for the participants’ integrity. Also during the analysis the interview texts have been carefully considered if interpretations are rooted in the participants’ narratives.

## Findings—the relationship to the care staff is ambivalent

In the naïve reading patients described it as unusual, embarrassing, impersonal, linked to shyness, and the feeling of powerlessness and causing inconvenience, to be dependent on help for care when admitted to an intensive care unit; shame, dignity and being violated seem to be pivotal. The care in the intensive care unit is described as good when meeting needs, without having to ask for help; this is perceived as nice as in this way you avoid causing inconvenience. The good nurse who is caring, kind, and human makes it easier to cope with the dependency of care. The participants stated positive experiences of care, but during interviews also reported experiences of violations. The importance of these is overruled which could be linked to a high degree of gratitude for having overcome serious disease.

In the structural analysis, the theme “The relationship to the care staff is ambivalent,” is expressed in the following sub-themes: “Being dependent on care can mean you are violated,” “The relation to staff is personal and caring” and “There is a deep gratitude linked to the dependency.”

### Being dependent on care can mean you are violated

For the participating patients the narratives of perceived dependency have led to stories about violation. The violation occurs when body and person are separated and the experience of integrity seems to be at stake. Narratives indicate violations as illuminated:

It was very transgressive when they touched me all the time. A lot of different people. No one, or maybe they did, but I didn't feel that anybody considered me as a person. It was just a body lying there that they had to get going again. And that was also fine because it meant that I survived but … (Participant 1)

The participant tells about the care which was experienced as instrumental and impersonal but accepted it as it had meant survival. It is indicated that the experience of integrity is at stake as distinctions are made between body and person.

The violation can also occur when the care received is not dignified. Patients gave reports of care where the nurse is experienced as cold and indifferent. The nurse is superficial and heavy-handed and she does not perform her work with empathy. One of the patients avoids asking the nurse for help and the patient postpones among other things the need for tracheal suctioning. The patient does not tell specifically about which feelings the experience has caused but she says: “… I will not treat other people in this way” and “This is the kind of person you don't forget.” Thus, the experience seems to have major importance and you could sense that the participant has experienced that her dignity has been violated as the care is perceived as being inhuman. Similar expressions show that the experience is accepted with the statement “… it was only a few times and the other (times) totally compensate for those.”

Finally, participants link the dependency on care several times to a crossing of a personal boundary for what is experienced as pleasant. One of the participants tells about episodes in connection with body care where “everything was just open” and where two hospital porters stand talking together in the room while the nurse cares for a pressure wound at the participant's lower back. The violations are thus a result of the exposure of the body but also of the way the staff act. When asking more about the experiences the participant expresses that it is only the exposure of the body which is perceived as uncomfortable and not the way the situations are managed as she fails to see how it could have been managed differently. There seems to be some ambivalence in the statement which could indicate that it is perceived as difficult for the participant to criticize the care. This could be the result of a high degree of gratitude, which is also expressed in the interview.

### The relation to staff is personal and caring

The exposure of the body is in itself violating but violation also seems to be dependent of the way staff reacts. The participating patients tell about good experiences with the care staff. They experience that their needs have been met in many situations without having to ask for help. At the intensive care unit you are spoiled and welcomed, the staff is exceptional and competent, and you feel well-informed. When the relationship to the staff is good, participants perceived being dependent as easier. Participants state that it seems to be of major importance that the relationship to the nurse is personal. It gives a sense of security and community.

The experiences indicate that doing everyday things with the nurses emphasized the good relation offering more than instrumental nursing. For instance illuminated in this situation:

Then I went to have a bath and it was, it is obviously a bit cold when the hot water is turned off; then imagine a nurse coming in with a blanket from a heated cupboard and throws it to me. You know what – right there I was walking on air. (Participant 2)

It is thus important to feel the nurse's consideration and to receive the care which goes beyond what is absolutely necessary. Participants also mention that you get a special relation to some of the nurses and to put it “it's as if you long for some of them (laughing)” tells how this patient has worked together with the staff and that she has been able to talk and relate to the nurses. The other participants did not experience having the same influence which could be explained by the fact that they had been sedated during a large part of their stay at the intensive care unit.

### There is a deep gratitude linked to dependency

Being dependent on care can mean you are violated. There seems to be some ambivalence in the narratives which could indicate that it is difficult to criticize the care because of a high degree of gratitude. The participating patients express gratitude for having recovered from critical illness and they talk about the nursing staff in an emotional way and in positive terms. The staff is referred to as admirable and great, illuminated as: “I could have hugged all of them” and “I would like to send a thank you and greeting to all.” The gratitude of having survived seems in some situations to mean that participants accept less positive experiences. It is difficult for participants to criticize the care they have received and it could be okay to be treated just as a body because it meant survival, “well you, you survive right? Damn it, is because of the people bustling about ….” Or illuminated as “I get annoyed with the staff because they don't understand what I was trying to communicate … I have absolutely nothing to complain about, nothing at all.”

Finally, there can be an element of humbleness in the gratitude, illuminated as:

I think you have to be happy that anybody would help you when you are in such a difficult situation … (Participant 3)

But when you cannot do it yourself and others would like to help you then you have to be grateful and say thank you and I have done that. (Participant 2)

It seems that you can get a feeling of being dependent and not being entitled to say “NO” or make demands to the care. The humbleness might be explained by the fact of difference in generations. Older patients come from a generation with a more pronounced belief in authorities. Moreover, it might play a role if a patient had had prolonged stays in the intensive care unit.

## Discussion

The interpreted whole with this study was that dependency is experienced as difficult, and the relationship with the nurses seems to be ambivalent. The good relationship is experienced to make dependency easier, whereas negative experiences make it harder to cope with dependency. The participants deal with dependency by accepting negative experiences in gratitude for having recovered from critical illness.

Being dependent could mean you are violated when you are an intensive care patient. The violation occurs with the exposure of the body and the way staff reacts. When body and person are separated and the patients do not receive respectful care and are e.g., exposed to inhuman instrumental nursing; integrity is at stake.

Martinsen (2012) writes about how encounters between people are supported by the united contradictions, openness, and the untouchable zone where you at the same time are present openly affected and evaluating the situation at a distance. The nurse's assessment makes it necessary to acknowledge the united contradictions and in the complimentarily of the contradictions, the vulnerable life is respected. Martinsen (2012) questions whether we are losing our ability to face these contradictions where life and its boundaries are respected. She questions whether we can manage to be in the openness with the vulnerable human being without seizing control of or invading it; in contrast to this, she describes how the skills of the time, which are classifying and fast can mean that the patient is objectified and the human being cannot been seen. Being objectified can be perceived as painful and could cause the human being to shut off. The nurse must be touched and moved to create a wondering reflection in relation to herself and the skills she has and in this way meet the vulnerable human being. Taking the starting point in Martinsen's thoughts, the violations described by participants could be interpreted as an expression of nurses not being able to be in the united contradictions, the untouchable zone and the openness. It could be a sign of distancing and lack of openness when a nurse is described as cold and indifferent. Moreover, it can be interpreted as a sign of excessive openness when personal care is performed without considering the boundaries of the patient's shyness.

Other studies have, similar to this study, described that patients experienced violations in connection with care. Delmar (2013) writes about admitted surgical patients who have been exposed to violations and about nurses who are indifferent, distancing, and thus patronizing in their approach. Within the intensive care context others, Almerud, Alapack, Fridlund, and Ekebergh (2007), McKinley et al. (2002) and Russell (1999), have reported findings indicating violations in connection with intimate care during the stay at the intensive care unit as the nurses “joke” and talk privately while the patients lie undressed. The experience has had such a great impact on some of the patients that they subsequently wished to be dead. McKinley et al. (2002) found that patients occasionally experience being objectified and that the care becomes impersonal, thus increasing the experience of being vulnerable. The patients’ positive experiences with the care staff are described as competent and the patients experience that their needs are covered, in many situations without having to ask for help. Considerate nurses offering more than just instrumental care provide a sense of community and a personal relation to the staff. When the relationship to the staff is good, dependency is described as being easier to contain. It is described that you obtain a special relation to some of the nurses. These findings are confirmed in several other studies at ICUs. Karlsson, Bergbom, and Forsberg (2012) describe how partnership and participation in care provide security for the patients, belief in getting better, and making it easier to overcome grief. McKinley et al. (2002) describe that good personal care and information reduce the experience of vulnerability. Samuelson (2011) describes that intensive care patients experience that good care and professional staff makes up for stressful experiences. Similarly, Wåhlin, Ek, and Idvall (2006) have also found that empowerment of the intensive care patient means supporting the patients is more security in some staff than in others.

It is meaningful to achieve a personal relationship to the staff and to be involved in one's own care. When the participants in this study get a feeling of being dependent and not being entitled to say no or make demands to the care; the feeling of powerlessness is at stake. Almerud, Alapack, Fridlund, and Ekebergh (2007) found that intensive care patients try to find out what it means to be a good patient and adapt to the routines of the system on the basis of this. One of the patients in the study experienced getting more attention and better care if the nursing staff liked him. In other contexts, Irurita and Williams (2001), Lomborg, Bjørn, Dahl, and Kirkevold (2005), Eriksson and Andershed (2008) and Delmar (2013) also found that patients, in a strategy to protect their own integrity, try to be good patients: not to make demands, not to complain or call too often, and to try to create a good relationship with the staff. This strategy is used when patients are conscious of their own vulnerability as a consequence of already compromised care.

It is interesting to transfer these thoughts to the intensive care context as the findings are similar to ours. Patients being patient and compliant in the interaction with staff could be an expression of an attempt to protect the integrity based on knowledge of their own vulnerability. We have not found an answer to why the feeling of powerlessness exists when meeting kind and welcoming staff. An explanation could be linked to experiencing lack of value and shame of being dependent on care. Moreover, it is a schism that our culture values self-dependence and independence (Henriksen & Vetlesen, 2000) and those patients at the same time experience that it is worthwhile to be compliant. In summary, the good caring nursing is important to the experience of being an intensive care patient and dependent and it can contribute to making dependency on care easier.

The participating patients express gratitude for having recovered from critical illness and they talk about staff in an emotional way and in positive phrases. Being grateful for having survived seems to mean that some participants accept less good experiences. To the oldest participants, humbleness also seems to be linked with gratitude. Gratitude is described in other studies on dependency on care, for example, in the study by Eriksson and Andershed (2008). They found that patients in their gratitude have the need to repay which is expressed through praising of the nursing staff. When participants in this study praise the nurses it might be because they wished to give something in return for the help they have received. Strandberg, Norberg, and Jansson (2003) have also found that patients defend the nurses when the care has been less satisfactory. Finally, Irurita and Williams (2001) found that patients try to justify reduced care to maintain their integrity which can threaten integrity further. The acceptance of the less good experiences expressed by participants can therefore be an attempt to try to maintain integrity.

## Conclusion

This study explores the perceived meaning of dependency on care as experienced by intensive care patients. Patients experience that it is difficult to be dependent on care, and their relationships to staff appears to be ambivalent. Good lived experiences with staff seem to make the dependency easier to accept. The care at the ICU is described as good and the personal relationship and being involved in one's own care, information, and communication seem to be of major importance for the lived experience of being dependent. Despite experiencing good care, the participants also tell about violations due to the nurses’ lacking ability to be in the field of tension between openness and the untouchable zone. The patients manage dependency by, for instance, accepting these violations and less good lived experiences as a consequence of the deep gratitude of having survived. Accepting bad experiences can be seen as an attempt to try to maintain integrity.

It seems relevant that nurses reflect on how the intensive care patient experiences dependency and highlight communication and patient involvement to a larger extent.

## Methodological considerations

Because of the recall problems it was very difficult to recruit participants, but with a phenomenological–hermeneutic approach it is important to delve into “the case itself.” The narratives from the three patients gave us rich in-depth knowledge of the perceived meaning of dependency and what is at stake when patients are dependent on care in the ICU.

The question of applicability to other ICU settings in Denmark is essential. The answer to the question of what counts as applicability will be that “generalizability” in qualitative research builds on recognizability and challenges to practice (Delmar, 2010). Recognizability appears by looking for communalities, similarities, and differences. But this can only form part of the “generalizability” of a finding; knowledge should be recognized and confirmed by others. Only when the recipient of new knowledge is able to relate it to his own practice, only then does it makes sense to him and the road is clear for understanding and practical application of the knowledge (Delmar, 2010).

This is a study with three participants which means that even though we have highlighted new knowledge, further research in the field of dependency in ICU settings has to be done.
